# Kaempferol attenuates liver fibrosis by inhibiting activin receptor–like kinase 5

**DOI:** 10.1111/jcmm.14528

**Published:** 2019-07-05

**Authors:** Taifu Xu, Shan Huang, Qianrong Huang, Zhiyong Ming, Min Wang, Rongrui Li, Yinnong Zhao

**Affiliations:** ^1^ Department of Hepatobiliary Surgery Affiliated Guangxi Tumor Hospital, Guangxi Medical University Guangxi China; ^2^ Department of General Surgery The Fourth Affiliated Hospital, Guangxi Medical University Guangxi China

**Keywords:** ALK5, hepatic stellate cells, kaempferol, liver fibrosis, Smad2/3

## Abstract

Liver fibrosis is a common public health problem. Patients with liver fibrosis are more likely to develop cirrhosis, or hepatocellular carcinoma (HCC) as a more serious consequence. Numerous therapeutic approaches have emerged, but the final clinical outcome remains unsatisfactory. Here, we discovered a flavonoid natural product kaempferol that could dramatically ameliorate liver fibrosis formation. Our data showed that intraperitoneal injection of kaempferol could significantly decrease the necroinflammatory scores and collagen deposition in the liver tissue. In addition, serum alanine aminotransferase (ALT), aspartate aminotransferase (AST), laminin (LN) and hyaluronic acid (HA) levels were significantly down‐regulated in kaempferol treatment group compared with those in the control group. Our study also demonstrated that kaempferol markedly inhibited the synthesis of collagen and activation of hepatic stellate cells (HSCs) both in vivo and in vitro. Furthermore, the results of Western blotting revealed that kaempferol could down‐regulate Smad2/3 phosphorylation dose‐dependently. These bioactivities of kaempferol may result from its targeted binding to the ATP‐binding pocket of activin receptor–like kinase 5 (ALK5), as suggested by the molecular docking study and LanthaScreen Eu kinase binding assay. Above all, our data indicate that kaempferol may prove to be a novel agent for the treatment of liver fibrosis or other fibroproliferative diseases.

## 1 INTRODUCTION

Liver fibrosis is the consequence of chronic or repeated liver injury caused by hepatotoxic agents such as alcohol, or chronic liver diseases such as alcoholic hepatitis, hepatic steatosis, viral hepatitis infection and autoimmune disorders.^1^ Chronic hepatic fibrosis progression ultimately results in cirrhosis, which is often associated with liver failure, portal hypertension and hepatocellular carcinoma (HCC).^2^ Although numerous therapeutic approaches have emerged and been reported to have promising effects, the actual clinical outcome remains unsatisfactory,^3^ mainly because of the fuzzy understanding about the mechanism underlying the development and progression of liver fibrosis. Therefore, it is an urgent task to uncover the mystery of how hepatic fibrosis develops and find more effective anti‐fibrotic agents for the treatment of liver fibrosis.

It is generally believed that dysfunction of hepatic stellate cells (HSCs) plays a major role in the process of hepatic fibrogenesis.^4^ HSCs are quiescent and function as vitamin A storage under normal physiological conditions.^5^ When HSCs are activated, they transdifferentiate into myofibroblast‐like cells, expressing alpha smooth muscle actin (α‐SMA) and secreting large amounts of collagen.^6^ When more collagen is accumulated, the normal liver parenchyma is replaced by scar tissue, eventually resulting in liver fibrosis. Among the profibrogenic cytokines, transforming growth factor‐β (TGF‐β) is the key cytokine which plays the major role during the fibrotic process.^7^ TGF‐β1 can bind to type II receptor and subsequently phosphorylate Smad2 and Smad3, which activates HSCs and triggers the transcription of pro‐fibrosis genes.^8^

Kaempferol is a natural flavonol that is abundant in broccoli, tea and other plants.^9^ Noticeably, this compound possesses a variety of biological and pharmacological activities, including antioxidant 10 and anti‐inflammatory activities,^11^ the inhibitory effect on tumour growth,^12^ and the therapeutic efficacy on inflammatory arthritis by preventing bone destruction.^13^ Hepatologically, kaempferol can protect normal liver cells against hydrogen peroxide (H_2_O_2_)‐induced cytotoxicity, ROS formation and DNA damage.^14^ Kaempferol could protect the liver against alcohol‐induced injury by attenuating the activity and expression of cytochrome 2E1.^15^ Some recent studies reported that kaempferol could attenuate airway epithelial‐mesenchymal transition (EMT), collagen deposition and fibrosis by modulating PAR1 activation.^16^ However, the effects of kaempferol on liver fibrosis and its associating molecular mechanisms are not well understood.

In the present study, we analysed the therapeutic efficacy of kaempferol in a carbon tetrachloride (CCl_4_)‐induced liver fibrosis model. Furthermore, the effects of kaempferol on different cellular behaviours of HSCs such as collagen secretion and activation were investigated. Lastly, we tried to uncover the molecular mechanisms that may be involved in the anti‐liver fibrosis effects of kaempferol.

## 2 MATERIALS AND METHODS

### 2.1 Establishment of the liver fibrosis model

A liver fibrosis model was induced by CCl_4_ in female C57BL/6 mice aged 6‐8 weeks (Shanghai Slac Laboratory Animal Company). All the animal procedures in this study were in accordance with the ethical standards and approved by the Committee on the Ethics of Animal Experiments of our institution.

The liver fibrosis model was generated according to a published protocol.^17^ In summary, CCl_4_ was first dissolved in 10% olive oil, and then, the mixture (0.5 mg/kg) was injected intraperitoneally (i.p.). The procedures were performed 3 times per week for 4 weeks. During this period, 20 mice were treated i.p. with kaempferol (10 μmol/L, 1 ml) daily, and additional 20 mice received an equal volume (1 ml) of the vehicle (sterile saline plus 10% DMSO) injection.

### 2.2 Serum biochemistry assays

Serum was extracted from the mouse blood by centrifugation (3000 g, 10 minutes). Laminin (LN) and hyaluronic acid (HA) were assayed by radioimmunoassay according to the manufacturer's instructions (North Institute of Biological Technology). Alanine aminotransferase (ALT) and aspartate aminotransferase (AST) concentrations were determined using an automatic biochemical analyzer (Hitachi Auto Analyzer 7170).

### 2.3 Histology and immunohistochemistry

Liver tissues were paraformaldehyde‐fixed overnight, paraffin‐embedded, sliced into 5‐μm thick sections, stained with haematoxylin and eosin (H&E) and Sirius red.

As for immunohistochemistry, sections were incubated with primary antibodies against collagen type I (Abcam, 1:200, ab34710) and α‐SMA (Abcam, 1:200, ab32575) overnight at 4°C. After incubation with HRP‐conjugated goat anti‐rabbit secondary antibodies (Maxim, KIT‐5005), the sections were counterstained with haematoxylin and developed with diaminobenzidine.

### 2.4 Cell culture

The mouse liver was perfused using the pronase/collagenase method, and primary HSCs were isolated by gradient centrifugation as described previously.^18^ HSCs were then cultured in Gibco™ Dulbecco's Modified Eagle Medium containing 10% foetal bovine serum (Gibco), supplemented with penicillin 100 U/mL and streptomycin 100 mg/mL. HSCs were incubated at 37°C in a humidified atmosphere with 5% CO_2_.

### 2.5 Quantitative real‐time PCR (qPCR)

Total RNA was isolated using TRIzol reagent (Invitrogen). Quantitative PCR amplification was performed with the ABI Prism 7900 system using SYBR Premix (Takara) according to the manufacturer's instructions. GAPDH was used as a reference gene. The primers used in this study were as follows: collagen, type‐I, alpha 1 (Col1a1), 5′‐GCTCCTCTTAGGGGCCACT‐3′ (forward), and 5′‐ATTGGGGACCCTTAGGCCAT‐3′ (reverse); α‐SMA, 5′‐ CCCAGACATCAGGGAGTAATGG‐3′ (forward), and 5′‐TCTATCGGATACTTCAGCGTCA‐3′ (reverse).

### 2.6 Western blotting

Cultured HSCs were lysed with RIPA buffer supplied with protease inhibitor cocktail (Roche). In brief, 20 μg protein was separated by 8% or 10% acrylamide gels and transferred onto polyvinylidene difluoride membranes. Then, the membranes were blocked with 5% bovine serum albumin for 1 hour. The separated proteins were immunodetected with the primary antibodies including the following: Col1a1 (Abcam, 1:500, ab34710), α‐SMA (Abcam, 1:500, ab32575), Smad2 (Cell Signaling Technology, 1:1000, #5339), Smad3 (CST, 1:1000, #9523), p‐Smad2 (CST, 1:1000, #18338), p‐Smad3 (CST, 1:1000, #9520), Smad6 (CST, 1:1000, #9519), Smad7 (Abcam, 1:1000, ab90086), Smad4 (CST, 1:1000, #38454), TGF‐β receptor I (CST, 1:1000, #3712) and TGF‐β receptor II (CST, 1:1000, #79424). After washing, the membrane was incubated with goat anti‐rabbit secondary antibody (CST, 1:2000, #7074) for 1 hour at room temperature, and the density of the protein bands was scanned by an Alpha Imager scanner (Alpha Innotech). ImageJ software was used for quantitative analysis of immunoreactive bands.

### 2.7 Immunofluorescence

HSCs were fixed with 4% paraformaldehyde for 20 minutes and blocked with 5% BSA for 1 hour and incubated with primary anti‐Smad2/3 (Abcam, 1:100, ab202445) overnight at 4°C, followed by the proper secondary antibody. The nuclei were stained with 4',6‐diamidino‐2‐phenylindole (DAPI) for 8 minutes. Fluorescence was analysed using a Zeiss 710 laser‐scanning microscope.

### 2.8 LanthaScreen Eu kinase binding assay for ALK5

We conducted LanthaScreen Eu kinase binding assay^19^ to analyse the interaction of kaempferol with ALK5. Kinase buffer used in this study consisted of 1 mmol/L ethylene glycol tetraacetic acid, 10 mmol/L MgCl_2_, 50 mmol/L HEPES [4‐(2‐hydroxyethyl)‐1‐piperazineethanesulfonic acid; pH = 7.5] and 0.01% Brij‐35. Kinase/antibody solution consisting of kinase tracer 178 (5 μl, 30 nmol/L, Invitrogen, PV5593), Eu‐anti‐GST antibody (6 nmol/L, Invitrogen, PV5594) and GST‐ALK5 (5 μl, 15 nmol/L, Invitrogen, PV5837) was mixed with 4‐fold serially diluted kaempferol (5 μl) and incubated in a low‐volume 384‐well plate (Corning Part #3676) for 1 hour at room temperature. Fluorescence signals were detected using a Tecan Infinite F‐500 plate reader. The emission ratio was calculated using the formula tracer emission (665 nmol/L)/antibody emission (615 nmol/L). IC_50_ was determined based on the sigmoidal dose‐response curve that was generated using GraphPad Prism software.

### 2.9 Statistical analysis

Statistical software package SPSS 20.0 (SPSS) was used for statistical analysis using ANOVA, with a post hoc least significant difference test. All values are expressed as mean ± standard deviation (SD). The difference between groups was regarded considerable at *P* < 0.05.

## 3 RESULTS

### 3.1 Kaempferol attenuates CCl_4_‐induced liver injury and inflammation

**Figure 1:**
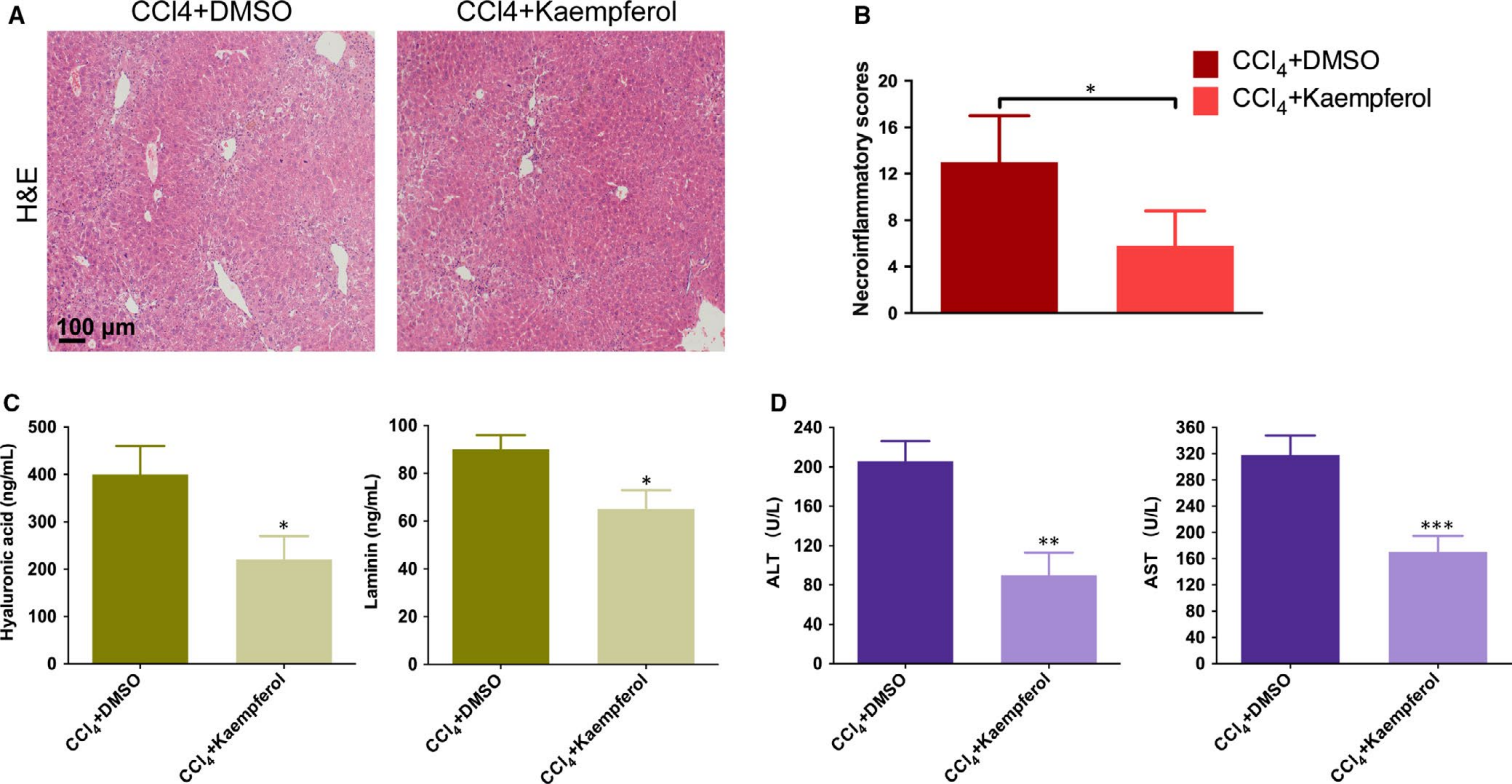
Kaempferol inhibits liver injury and inflammation. A, Images of H&E‐stained liver sections after application with DMSO or kaempferol (10 μmol/L). Scale bars, 100 μm. B, Quantification of necroinflammatory scores. C, Serum levels of hyaluronic acid (HA) and laminin (LN) and (D) serum levels of alanine aminotransferase (ALT) and aspartate aminotransferase (AST) after application with DMSO or kaempferol (10 μmol/L). n = 20/20 (20 mice in DMSO and kaempferol group, respectively). All experiments were carried out three times to assess reproducibility. Data are demonstrated as mean ± SD. *P < 0.05; **P < 0.01; ***P < 0.001

To investigate the anti‐fibrosis effect of kaempferol in vivo, we used CCl_4_‐induced mouse models. In this model, highly reactive metabolites were produced, which caused liver toxicity and further resulted in severe injury to hepatocytes and subsequent liver fibrosis.^20^ Histological analyses demonstrated that the percentage area of necrotic regions and the number of inflammatory cells surrounding the centrilobular veins were dramatically increased after repeated injections of CCl_4_ (Figure 1A). Conversely, in the kaempferol‐treated group, these histological changes were significantly ameliorated (Figure 1A). Furthermore, the necroinflammatory score decreased significantly after i.p. injection of kaempferol, as indicated by the Ishak‐modified HAI index (Figure 1B).

Serum HA and LN are two effective indicators reflecting the extent of liver fibrosis.^21^ As shown in Figure 1C, HA and LN levels in mice receiving i.p. injection of kaempferol were significantly lower than those in DMSO‐treated mice. In addition, serum ALT and AST levels were down‐regulated markedly after kaempferol treatment as compared with DMSO control (Figure 1D). The above results indicate that kaempferol could effetely attenuate CCl_4_‐induced liver injury and inflammation.

### 3.2 Kaempferol suppresses HSCs collagen synthesis both in vitro and in vivo

**Figure 2:**
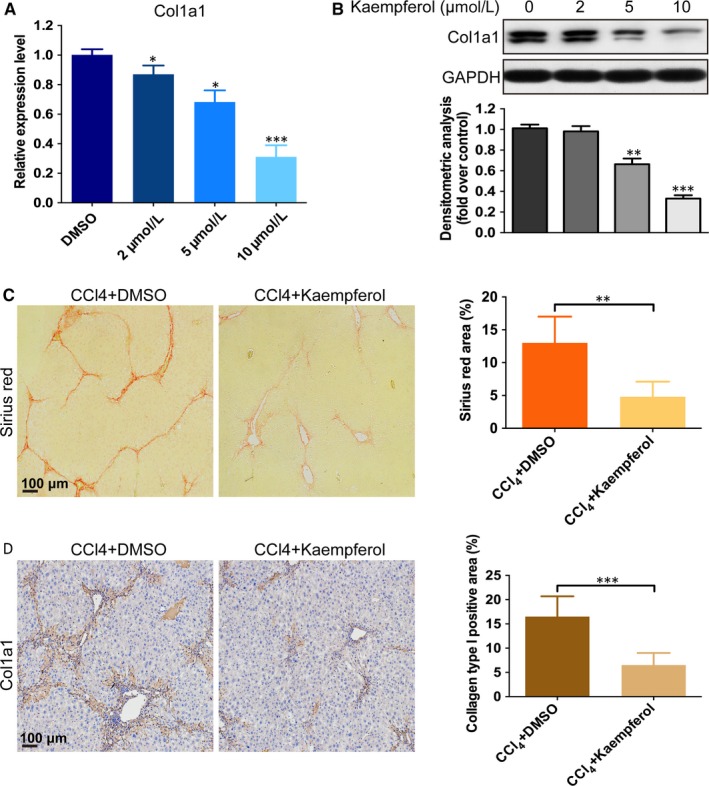
Kaempferol suppresses HSCs collagen synthesis. A, The mRNA (incubate 3 d) and B, protein (incubate 5 d) levels of Col1a1 in HSCs after incubation with DMSO or kaempferol. n = 3. C, Sirius red staining and quantification of the percentage of Sirius red‐positive area. Scale bars, 100 μm. n = 20/20 (20 mice in DMSO and kaempferol group, respectively). D, Immunohistochemistry images of Col1a1 and quantification of the percentage of Col1a1‐positive area. Scale bars, 100 μm. n = 20/20 (20 mice in DMSO and kaempferol group, respectively). All experiments were carried out 3 times to assess reproducibility. Data are demonstrated as mean ± SD. *P < 0.05; **P < 0.01; ***P < 0.001

Knowing that extracellular matrix deposition plays a crucial role in the process of liver fibrosis formation,^1^ we investigated whether kaempferol could inhibit collagen synthesis. After incubation with kaempferol for 3 days, the mRNA expression of Col1a1 in HSCs was down‐regulated markedly in a dose‐dependent manner (Figure 2A). Nevertheless, the protein level of Col1a1 also decreased, which was similar to the change in mRNA level (Figure 2B). We further performed Sirius red staining and immunohistochemistry staining to investigate the effect of kaempferol on collagen production in vivo. Repeated injections of CCl_4_ caused significant collagen accumulation in the mouse model (Figure 2C, 2). In contrast, the mice receiving kaempferol injections showed significantly lower collagen deposition and more preserved hepatic parenchyma than the DMSO‐injection group (Figure 2C, 2).

### 3.3 Kaempferol inhibits HSCs activation both in vitro and in vivo

**Figure 3:**
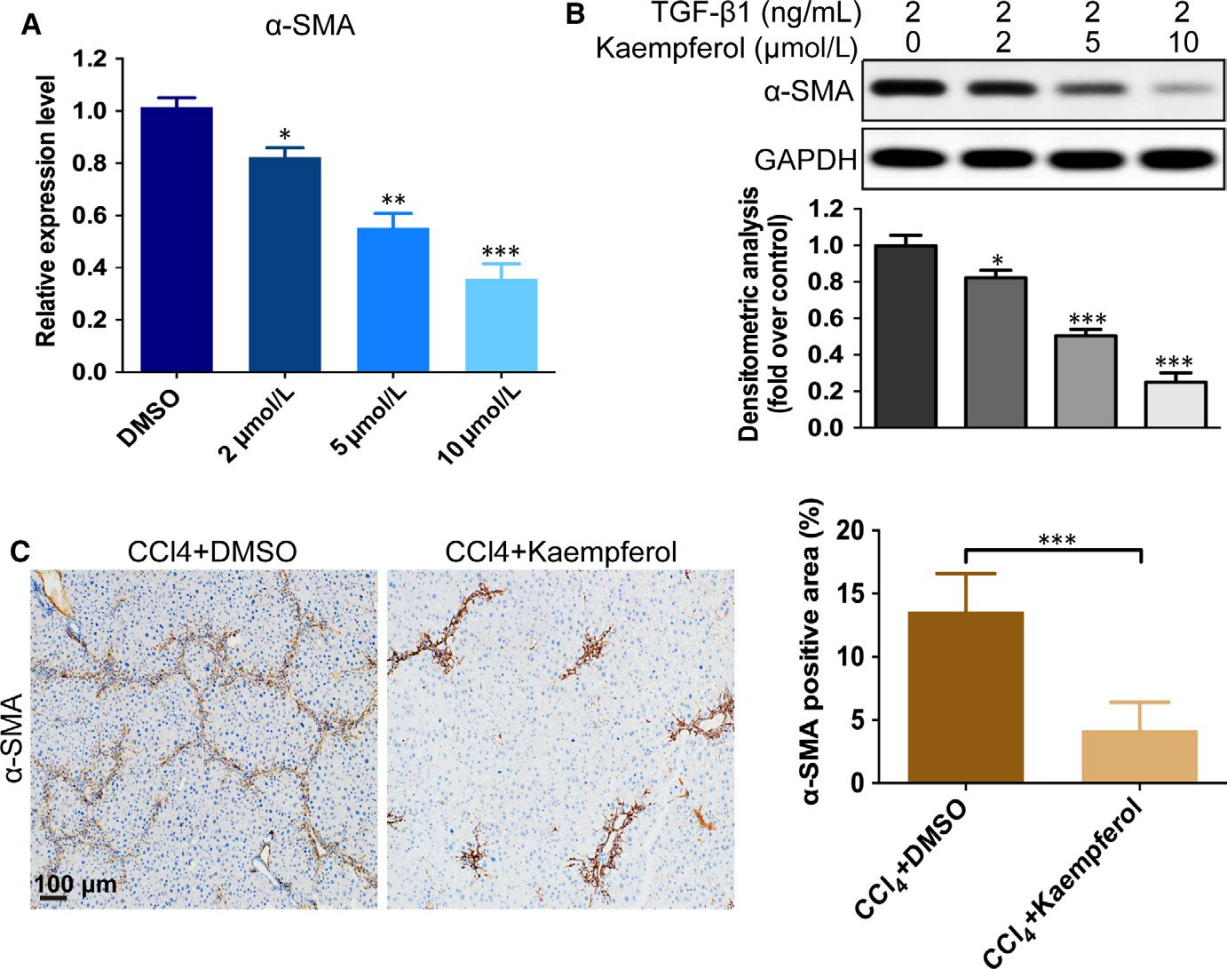
Kaempferol inhibits HSCs activation. A, The mRNA (incubate 3 d) and B, protein (incubate 5 d) levels of α‐SMA in HSCs after incubation with TGF‐β1 (2 ng/mL) and DMSO or kaempferol. n = 3. C, Immunohistochemistry images of α‐SMA and quantification of the percentage of α‐SMA‐positive area. Scale bars, 100 μm. n = 20/20 (20 mice in DMSO and kaempferol group, respectively). All experiments were carried out 3 times to assess reproducibility. Data are demonstrated as mean ± SD. n = 20/20, *P < 0.05; **P < 0.01; ***P < 0.001

As the activation of HSCs is the essential factor in liver fibrosis development,^22^ we investigated whether kaempferol could inhibit HSCs activation. The result demonstrated that both the mRNA and protein levels of α‐SMA induced by TGF‐β1 were down‐regulated markedly after kaempferol treatment (Figure 3A,B). Additionally, we detected the effect of kaempferol on the expression of α‐SMA in vivo. As shown in Figure 3C, the expression of α‐SMA was significantly lower in kaempferol‐injected mice than that in DMSO‐injected group (Figure 3C). These results indicated that kaempferol could effectively inhibit HSCs activation in the CCl_4_‐induced mouse model and in HSCs induced by TGF‐β1.

### 3.4 Kaempferol down‐regulates the phosphorylation of Smad2 and Smad3

**Figure 4:**
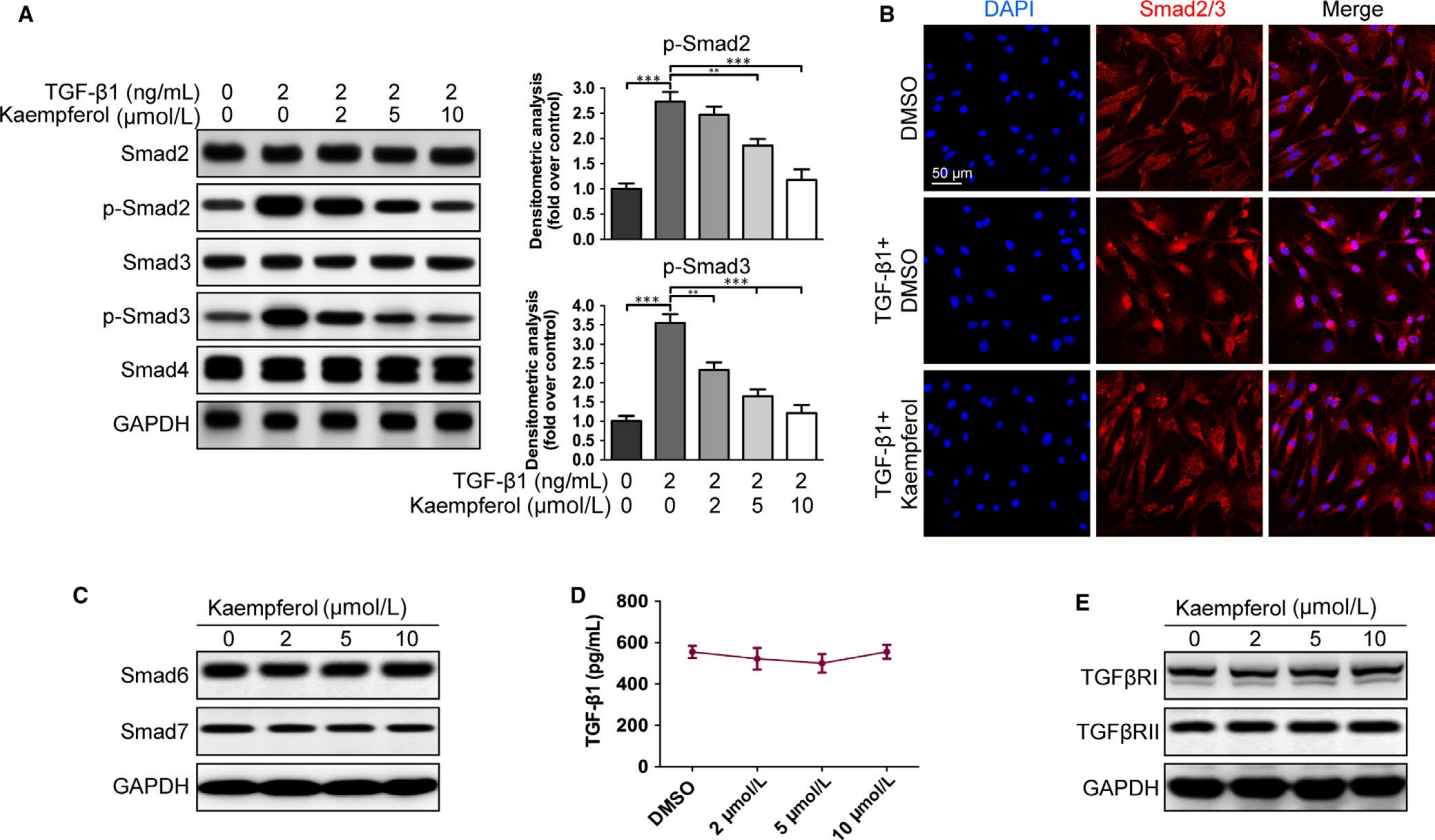
Kaempferol reduces Smad2 and Smad3 phosphorylation. A, Levels of Smad2, p‐Smad2, Smad3, p‐Smad3 and Smad4 in HSCs pretreated with TGF‐β1 (2 ng/mL) and kaempferol at different concentrations (2‐10 μmol/L) were detected using Western blotting and thereafter quantified. n = 3. B, Immunofluorescence images exhibit a significant reduction in TGF‐β1‐induced Smad2/3 translocation into the nuclei. Smad2/3 is shown by red fluorescence. Nuclei were stained with DAPI and shown by blue fluorescence. Scale bars, 50 μm. n = 3. C, Levels of Smad6 and Smad7 after HSCs were incubated with kaempferol or DMSO. n = 3. D, Kaempferol has no significant impact on the expression of TGF‐β1 based on ELISA assay. n = 3. E, Levels of TGF‐β receptor I and II with kaempferol incubation at different concentrations. n = 3. All experiments were carried out 3 times to assess reproducibility. Data are demonstrated as mean ± SD. **P < 0.01; ***P < 0.001

During the fibrotic process, TGF‐β1 is the key regulating cytokine, and was reported to have essential impact on liver fibrosis.^23^ In TGF‐β1 signalling, Smads proteins are major mediators via receptor‐induced phosphorylation and nuclear translocation.^24^ Based on these findings, we next investigated the TGF‐β1/Smads pathway. As shown in Figure 4A, the TGF‐β1‐induced (2 ng/mL) phosphorylation of Smad2 and Smad3 was down‐regulated dose‐dependently in HSCs after incubation with kaempferol (2‐10 μmol/L) for 12 hour. In addition, immunofluorescence results found that kaempferol (10 μmol/L) treatment could markedly inhibit TGF‐β1‐induced (2 ng/mL) translocation of Smad2/3 into the nucleus (Figure 4B). To further investigate which step in TGF‐β1/Smads pathway was changed by kaempferol, we first detected the effect of kaempferol on Smad6 and Smad7, two negative regulators in the TGF‐β1/Smads pathway.^25^ Unfortunately, neither of them was affected by kaempferol (Figure 4C). Next, we performed ELISA to detect whether kaempferol could inhibit TGF‐β1 secretion in HSCs. The results showed that TGF‐β1 expression was similar in both kaempferol‐ and DMSO‐treated cells (Figure 4D). Additionally, the protein levels of TGF‐β receptors I and II were also tested and no obvious effect was observed (Figure 4E).

### 3.5 Kaempferol binds selectively to the ATP‐binding site of activin receptor–like kinase 5

**Figure 5:**
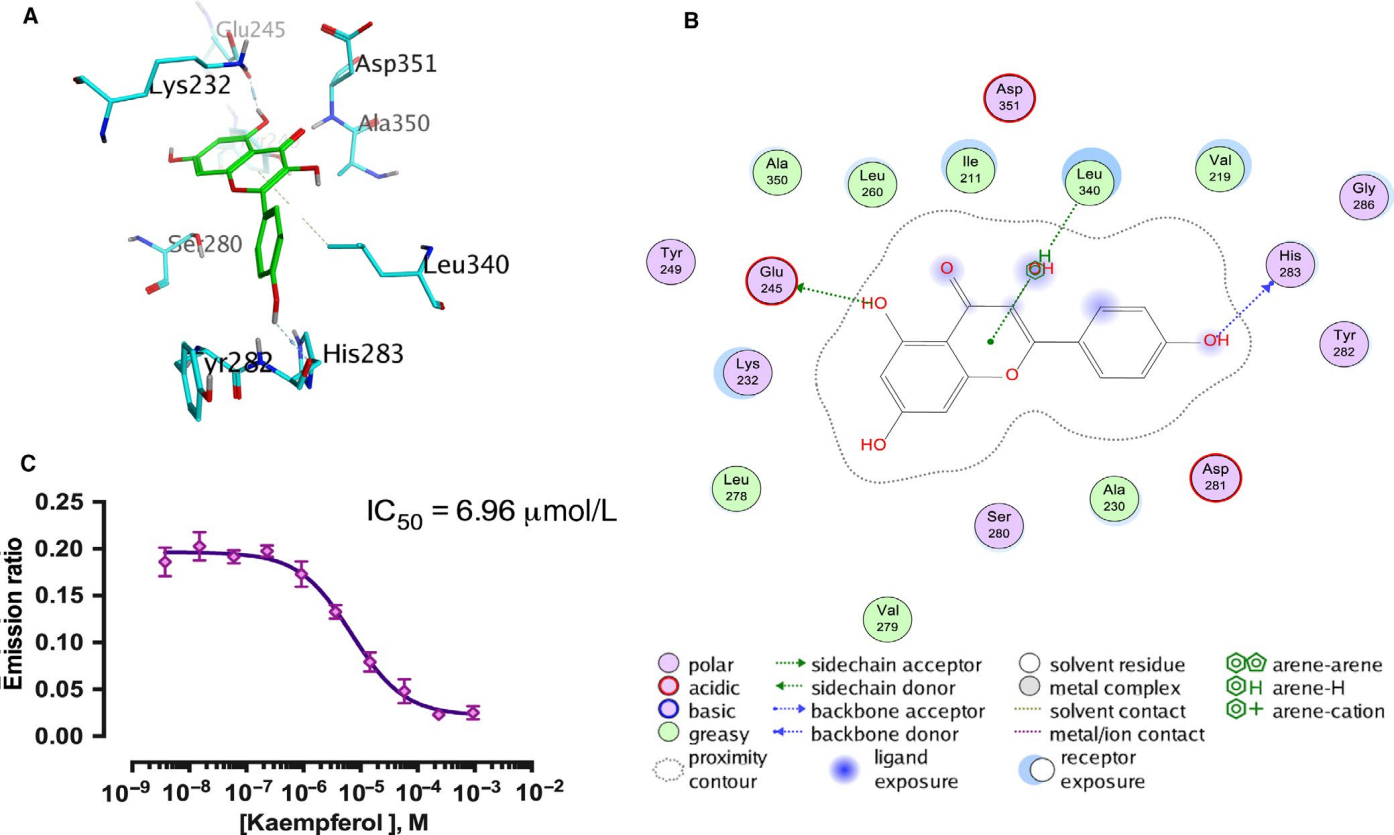
Kaempferol selectively binds to ALK5. A and B, A schematic diagram of computational docking simulation of kaempferol interaction with the ATP‐binding site of ALK5. Amino acid residues interacting with the compounds are indicated, and hydrogen bonds are represented with straight dashed lines. C, Dose‐response curve of LanthaScreen Eu kinase binding assay demonstrated a dose‐dependent decrease in emission ratios induced by kaempferol. The IC_50_ value was 6.96 μmol/L. n = 3. All experiments were carried out 3 times to assess reproducibility. Data are demonstrated as mean ± SD

Knowing that Smad2 and Smad3 are phosphorylated by the Serine/Threonine kinase domain by binding to the specific receptor TGFβRI (also known as ALK5) during the transduction of the TGF‐β1/Smads pathway,^26^ we explored whether kaempferol could inhibit the phosphorylation of Smad2 and Smad3 by direct binding to the catalytic region of ALK5 using the computational molecule docking study. The 3D interaction map showed that the 5‐hydroxyl of kaempferol could form a hydrogen bond with the Glu245, and the 4'‐hydroxyl could form another hydrogen bond with His283 of the ATP‐binding site of ALK5 (Figure 5A, 5). In addition to the two hydrogen bonds, an H‐π interaction formed between the flavone core and Leu340 (Figure 5A,B). Furthermore, we performed a LanthaScreen Eu kinase binding assay to further justify our hypothesis. The sigmoidal dose‐response curve showed that the emission ratio was dose‐dependently decreased by kaempferol and the IC_50_ value was calculated as 6.96 μmol/L (Figure 5C). These data indicate that kaempferol could selectively bind to the ATP‐binding site of ALK5.

## 4 DISCUSSION

Liver fibrosis is a complex fibroproliferative disorder caused by persistent inflammatory responses, usually secondary to various pathogenic factors, such as alcohol, virus, hepatotoxic medications and autoimmune response.^27^ Ultimately, progressive liver fibrosis progresses to cirrhosis. Other than liver transplantation, no effective treatment is currently available.^28^ Hence, finding effective ways to attenuate liver fibrosis is the essential step for preventing liver cirrhosis and failure.^29^

In the present study, we found that i.p. administration of kaempferol could significantly decrease the area of necrotic regions and down‐regulate the serum levels of HA, LN, ALT and AST in a mouse model of liver fibrosis induced by CCl_4_. The results showed that kaempferol could protect the liver against fibrosis in vivo. Overabundant collagen accumulation is known to be a typical histopathological characteristic of liver fibrosis.^30^ We found that kaempferol could not only suppress collagen type I expression in HSCs but reduce collagen density in the liver tissue. The findings were similar to the effect of kaempferol treatment in a cardiac fibrosis mouse mode, in which researchers found that collagen type I and type III were markedly reduced after kaempferol treatment.^31^ HSCs play a key role in liver fibrosis formation. Activated HSCs express α‐SMA and possess contract abilities.^32^ Our study revealed that kaempferol remarkably attenuated α‐SMA expression in the mouse model induced by CCl_4_ and further inactivated HSCs stimulated by TGF‐β1. Similar to our findings, Gong *et al* pointed out that kaempferol treatment reduced α‐SMA expression in an endotoxin‐induced airway fibrosis mouse model.^16^

TGF‐β1 is the key mediator during the process of liver fibrosis and widely implicated in HSC activation, HSC proliferation and extracellular matrix (ECM) production.^33^ Our results indicated that kaempferol down‐regulated the level of phosphorylated Smad2 and Smad3 in a dose‐dependent manner. In addition, the protein levels of Smad6/7, TGF‐β1 and TGF‐β receptors were not significantly altered. Researchers have shown interactions between a number of compounds and ALK5, and this kind of affinity has been confirmed to be associated with inactivation of TGF‐β/Smads signalling.^34^, ^35^ The data obtained from our computational molecule docking study and LanthaScreen Eu kinase binding assay showed that kaempferol could selectively bind to the ATP‐binding pocket of ALK5. As the ATP‐binding pocket is the core structure of the catalytic domain of ALK5, this interaction could, at least partially, explain the down‐regulation of TGF‐β1/Smad2/3 signalling after kaempferol intervention. Similarly, small interfering RNAs (siRNAs) could also target specific proteins such as VEGF,^36^ MMP‐2 37 and Foxf1 38 to attenuate extracellular matrix deposition and liver fibrosis formation. However, how to delivery siRNAs into targeted cells effectively and efficiently remains to be a problem that needs to be solved.^39^ The small‐molecular‐weight kaempferol described in the present study showed strong lipid solubility and strong permeability without needing a delivery vehicle.

In conclusion, our results demonstrate that the natural molecule kaempferol could effectively attenuate liver fibrosis formation, inhibit HSCs activation and further suppress HSCs collagen synthesis both in vivo and in *vitro*. In addition, kaempferol could selectively bind to ALK5 and further down‐regulate the TGF‐β1/Smads pathway. Kaempferol may prove to be an anti‐fibrosis agent for liver fibrosis or other fibrotic diseases.

## CONFLICTS OF INTEREST

The authors confirm that there are no conflicts of interest.

## AUTHORS' CONTRIBUTIONS

Taifu Xu and Yinnong Zhao designed the study. Shan Huang and Qianrong Huang contributed to the data collection. Zhiyong Ming, Min Wang and Rongrui Li performed the data analysis and interpretation. Taifu Xu and Shan Huang wrote the manuscript; Yinnong Zhao contributed to the critical revision of article. All authors read and approved the final manuscript.

## Data Availability

The data that support the findings of this study are available from the corresponding author upon reasonable request.
